# Notes and News

**Published:** 1900-07-07

**Authors:** 


### Notes and News.

A convalescent home for thirty-two female patients has been opened at Ilkley by the Leeds Workpeople's Hospital Fund. An excellent house and grounds have been secured for the sum of £2,910. The furnishing cost between £700 and £800.

The Earl of Pembroke distributed the prizes and medals to the students of Guy's Hospital Medical School on Wednesday. A garden party was held on the occasion, and the new laboratories, wards, and museums were open for inspection. Many of the guests, as well as those connected with the college, wore academic costume.

According to the *Medical News*, a New York paper be it observed, a post office inspector recently invaded the Metropolitan Medical College in West Van Buren Street, Chicago, and arrested four of its officers—its president, vice-president, and two secretaries. The institution is also known as the Independent Medical College, and the evidence goes to show that it is a mere "diploma mill," its faculty selling "degrees" for the practice of medicine and law at prices ranging from 3 to 200 dollars. It is stated that the fraud is an extensive one, and that the "graduates" of this college are practising in every State, and even abroad.

The present impasse in regard to the Swansea Hospital Fête forms an object-lesson as to the actual benefit resulting from entertainments as a means of increasing the revenues of charitable institutions. The inner workings are not always published, and the public remains in ignorance of the enormous percentage of the proceeds which frequently go towards expenses. In many cases people patronise an entertainment which they do not care for, under the impression they are largely helping a charity; and they would be much distressed to find that, perhaps, only one half the amount given goes to the object they are interested in. On the other hand, there are some who always require a *quid pro quo*. To these a charity entertainment is an advantage, as they gain amusement while doing good. The truly charitable have better ways of helping the needy.

The Asylums Board will borrow £75,000, at 3½ per cent. interest, from the County Council for the purpose of erecting an asylum at Tooting Bec.

The Conference on Malaria, to be held in Liverpool at the end of July, has been postponed owing to other medical fixtures having been made for the same date.

Hospital Saturday in London is fixed for October 13th this year. At the last meeting of the Board the Secretary reported an increase in the collection from workshops and business houses of £143 10s. during the present year.

The Commissioners in Lunacy have prepared a draft clause to be inserted in the Lunacy Amendment Bill now before Parliament, dealing with practices such as have been revealed in connection with the St. Pancras Board of Guardians and the traffic with the owners of private asylums.

Last week the Princess Christian opened the new wing which has been added to the Croydon General Hospital in commemoration of the Diamond Jubilee. The new wing, which will accommodate forty patients, has been erected at a cost of £11,000. Of this sum £6,000 was subscribed.

A Memorial Hospital for Tropical Diseases is to be founded in honour of the late Miss Mary Kingsley, and to be called after her name. Mr. A. L. Jones, the chairman of the Liverpool School for Tropical Diseases, has headed a subscription list with £1,000; Mr. Blaize, of Lagos, has given £500, and other promises have been made.

Professor Finsen's treatment for lupus and some other diseases of the skin by light is now in full operation at the London Hospital. The apparatus necessary was presented by the Princess of Wales. Poor patients are treated gratuitously in the morning, and in the afternoon the well-to-do can receive treatment by payment.

The meeting of the Metropolitan Asylums Board at the new offices on the Thames Embankment was made the occasion of a presentation to Sir Edwin Galsworthy, the chairman. The Hon. G. Talbot, M.P., in presenting the congratulatory address in a gold casket, mentioned the fact that Sir Edwin had been chairman of the Board for nearly 19 years, and a member of it for more than 32 years. He pointed out the progress and extension of the operations of the Board since the commencement of the chairman's term of office. He bore testimony also to the high qualities exercised by Sir Edwin in the performance of his duties.

The work of the London School of Tropical Medicine is already beginning to show itself. Mr. G. Low, a student of the school, is the first Craggs' scholar, and is, in conjunction with Dr. Sambon, making experiments in the Roman Campagnia. The scholarship, which has been founded by Mr. J. G. Craggs, one of the honorary secretaries of the Prince of Wales's Hospital Fund, is a valuable encouragement to the study of malarial disease.

